# Serum leptin levels are positively associated with aortic stiffness in patients with chronic kidney disease stage 3–5

**DOI:** 10.1080/21623945.2020.1764799

**Published:** 2020-05-13

**Authors:** Jing-Wun Lu, Po-Jui Chi, Yu-Li Lin, Chih-Hsien Wang, Bang-Gee Hsu

**Affiliations:** a School of Medicine, Tzu Chi University, Hualien, Taiwan; b Division of Nephrology, E-DA Hospital, E-DA Cancer Hospital, I-Shou University, Kaohsiung, Taiwan; c Institute of Medical Sciences, Tzu Chi University, Hualien, Taiwan; d Division of Nephrology, Hualien Tzu Chi Hospital, Buddhist Tzu Chi Medical Foundation, Hualien, Taiwan

**Keywords:** Leptin, carotid–femoral pulse wave velocity, aortic stiffness, chronic kidney disease, age, systolic blood pressure, diabetes mellitus

## Abstract

Leptin potentially exerts atherogenic effects.This study evaluated the relationship between serum leptin levels and aortic stiffness in patients with stage 3–5 chronic kidney disease (CKD). Totally 205 participants were enrolled. Fasting blood sample were checked and serum leptin were measured by enzyme immunoassay. Aortic stiffness was measured as the carotid–femoral pulse wave velocity (cfPWV). 73 (35.6%) of 205 patients showed cfPWV >10 m/s were defined as aortic stiffness group. Compared with the remaining patients, the aortic stiffness group had high prevalence of diabetes mellitus, older age, higher waist circumference, body fat mass, systolic blood pressure, fasting glucose, and higher serum leptin level. In multivariable logistic regression analysis the independent predictors of cfPWV >10 m/s included leptin levels (odds ratio [OR]: 1.061, 95% confidence interval [CI]: 1.027–1.095, *P* < 0.001), age (OR: 1.064, 95% CI: 1.033–1.096, *P*< 0.001), and systolic blood pressure (OR: 1.021, 95% CI: 1.006–1.037, *P* = 0.006). Multivariable forward stepwise linear regression analysisshowed a positive association between log-transformed leptin levels and log-cfPWV (β = 0.192, adjusted R^2^ change = 0.042, *P* = 0.001). Thus, aortic stiffness is positively correlated with serum leptin levels in patients with stage 3–5 CKD.

**Abbreviations:** BUN, blood urea nitrogen; cfPWV, carotid–femoral pulse wave velocity; CI, confidence interval; CKD, chronic kidney disease; Cre, creatinine; DBP, diastolic blood pressure; DM, diabetes mellitus; eGFR, estimated glomerular filtration rate; LDL-C, low-density lipoprotein cholesterol; OR, odds ratio; SBP, systolic blood pressure; TCH, total cholesterol; TG, triglycerides

## Background

Cardiovascular disease is the primarily aetiology of mortality in chronic kidney disease (CKD) patients[1]. Many clinical researches have reported poor kidney function and proteinuria are significantly related with cardiovascular disease, hospitalization, and mortality, independent of the traditional risk factors[2]. Aortic stiffness – a consequence of ageing – is an important determinant of cardiovascular risk, which affects conduit function, reduces end organ perfusion, and increases the risk of ischaemic heart disease and stroke [3–5]. A recent study reported that arterial stiffening was associated with impaired renal function in CKD and that it was predictive of the progression of the kidney disease and of the patient’s cardiovascular outcome[6]. Carotid–femoral pulse wave velocity (cfPWV) is the gold standard for evaluating arterial stiffness; this method is non-invasive, and cfPWV has been shown to be an important predictor of cardiovascular events, cardiovascular mortality, and even all-cause mortality [7,8].

Leptin, mainly produced by adipose tissue, is a 167-amino acid peptide hormone. It is primarily involved in the regulation of whole body energy homoeostasis and metabolism. Recent studies have suggested its role in increased cardiovascular risk associated with obesity. [9,10] Acting via leptin receptors in many tissues, leptin can induce endothelial dysfunction, stimulate systemic inflammation, elevate oxidative stress, and increase vascular smooth muscle hypertrophy; it may, therefore, play an active role in atherosclerosis[11]. Therefore, the clinical implication of hyperleptinemia had been linked with cardiovascular outcomes in patients with coronary artery disease, stroke, carotid artery disease, peripheral artery disease, chronic kidney disease, and type 2 diabetes mellitus (DM) [12,13]. However, studies of the relationships between leptin levels and cardiovascular risk have mainly performed on DM or obesity patients. The role of leptin on arterial stiffness in CKD patients is still not well studied. Thus, our aim was to investigate the association between serum leptin levels and cfPWV in CKD stage 3–5 patients.

## Subjects and methods

### Participants

Initially, 230 CKD participants enrolled, 25 participants were excluded on account of malignancy (n = 3), a chronic inflammatory disease (n = 3), heart failure (n = 5), or chronic obstructive pulmonary disease (n = 4), or if they refused to provide informed consent for participation (n = 10). Finally, a total of 205 patients with CKD over 18 years old without kidney transplantation or dialysis before were enrolled from the nephrology outpatient department at our medical centre in Hualien, Taiwan, between January and December 2016. All the participants were educated with detailed CKD care, focused on the salt and protein restriction, and the avoidance of nephrotoxins. This study was proved by the Protection of Human Subjects Institutional Review Board at Tzu Chi University and Hospital (IRB103-136-B), and informed consent was written before participation.

Estimated glomerular filtration rates (eGFRs), determined by using the Chronic Kidney Disease Epidemiology Collaboration equation, measured at least 3 months apart and used the mean value[14]. All patients were divided into different stage CKD according to the Kidney Disease Outcomes Quality Initiative criteria[15]. Patients were considered as CKD stage 3 as eGFR = 59–30 ml/min per 1.73 m^2^, stage 4 as eGFR = 29–15 ml/min per 1.73 m^2^, and stage 5 as eGFR <15 ml/min per 1.73 m^2^, separatively. All the participants received SBP and DBP measurement; check in the morning by using a standard mercury sphygmomanometer on right arm. Before BP measurement, they had been seated for at least 10 min. Patients who used any anti-hypertensive medications over 2 weeks, SBP ≥140 mmHg, and/or DBP ≥90 mmHg are considered hypertension disease. Either fasting plasma glucose ≥126 mg/dl or usage of oral hypoglycaemic medications or insulin is defined as DM[16].

### Anthropometric and biochemical measurements

Participants were measured body weight, height, and waist circumference in light-weight clothing without shoes. Body mass index was obtained by using the body weight (kg) divided by the height (m) squared. Body fat mass was measured by bioimpedance analysis (Biodynamic-450; Biodynamics Corporation, Seattle, WA, USA) [17–20].

Blood sample (approximately 5 ml) was taken after 8 hours fasting in the morning and centrifuged for 10 min at 3000 × *g* immediately. Serum levels of total calcium, phosphorus, blood urea nitrogen (BUN), creatinine (Cre), fasting glucose, total cholesterol (TCH), triglycerides (TG), and low-density lipoprotein cholesterol (LDL-C) were measured using an autoanalyzer (Siemens Advia 1800; Siemens Healthcare GmbH, Erlangen, Germany). Serum leptin were measured by enzyme immunoassay with commercial kit (SPI-Bio, Montigny le Bretonneux, France) [17–20].

### cfPWV measurements and definition of aortic stiffness

cfPWV measurement was done as previously described by applanation tonometry using a SphygmoCor system (AtCor Medical, West Ryde, Australia) [17–20]. Participants had rested for at least 10 min in supine position and then received the measurement. Pulse wave were detected by carotid and femoral measurement sites. Time difference (Δt) was obtained. The carotid-femoral distance obtained by subtracting the carotid measurement site to sternal notch distance from the sternal notch to femoral measurement site distance (d). cfPWV (m/s) was calculated by divided the distance with time difference (d/Δt). Patients with cfPWV >10 m/s were sorted into the aortic stiffness group, according to the guidelines of the European Society of Hypertension and European Society of Cardiology[8]. Others were included in the control group.

### Statistical analysis

The Kolmogorov–Smirnov test was used to check the normality of data distributions. We expressed normally distributed data as means ± standard deviation, and evaluated comparisons between groups by using two-tailed Student’s independent t-tests. We expressed non-normally distributed data as medians and interquartile ranges, and evaluated comparisons by using the Mann–Whitney U test. Data based on numbers of patients were expressed as the percentage (%) f study population, and evaluated by the chi-square test. Variables that were significantly associated with the aortic stiffness group were further tested for independence by multivariable logistic regression analysis. Because cfPWV, TG, fasting glucose, BUN, Cre, and leptin were not normally distributed, we had transformed the collected data to base 10 logarithmic values to achieve normality. Variables that were significantly correlated with log-cfPWV were tested for independence in a simple linear regression analysis. Variables independent in simple linear regression were then adjusted in a multivariable forward stepwise regression analysis. A *P* value <0.05 was considered to be statistically significant. The statistical analyses were performed with SPSS for Windows version 19.0 (SPSS, Chicago, IL, USA) package SPSS for Windows (Version 19.0, SPSS Inc., Chicago, IL, USA).

## Results

Comorbid conditions of the 205 participants included DM (n = 95; 46.3%), hypertension (n = 170; 82.9%). Table 1 compares baseline clinical characteristics between the aortic stiffness group (n = 73; 36%) and the remaining participants (the control group; n = 132; 64%). No statistically significant difference was found in sex ratio, hypertension, diabetes mellitus or chronic glomerulonephritis or in terms of use of statins, fibrates, or anti-hypertensive drugs between the two groups. However, higher prevalence of DM in the aortic stiffness group was noted (*P* = 0.007). The aortic stiffness group were older (*P* < 0.001) and had higher serum leptin level (*P* < 0.001), systolic blood pressure (SBP, *P* = 0.001), waist circumference (*P* = 0.012), body fat mass (*P* = 0.025), and fasting glucose (*P* = 0.049) than the control group.

**Table 1.** Clinical variables of the 205 chronic kidney disease patients with or without aortic stiffness

Characteristics	All Patients (*n* = 205)	Control Group (*n* = 132)	Aortic Stiffness Group (*n* = 73)	*P* value
Age (years)	69.07 ± 13.43	66.08 ± 14.09	74.48 ± 10.20	< 0.001*
Height (cm)	158.78 ± 8.79	158.90 ± 8.72	158.57 ± 8.99	0.794
Body weight (kg)	66.35 ± 14.31	65.95 ± 14.53	67.08 ± 13.99	0.592
Body mass index (kg/m^2^)	26.18 ± 4.45	25.99 ± 4.62	26.52 ± 4.14	0.413
Waist circumference (cm)	87.24 ± 10.95	85.83 ± 11.07	89.81 ± 10.32	0.012*
Body fat mass (%)	28.83 ± 8.39	27.86 ± 8.81	30.58 ± 7.32	0.025*
cfPWV (m/s)	9.00 (7.25–11.20)	7.80 (6.63–8.98)	12.40 (10.90–14.70)	< 0.001*
SBP (mmHg)	149.40 ± 24.44	145.14 ± 23.67	157.12 ± 24.07	0.001*
DBP (mmHg)	83.59 ± 12.82	83.04 ± 12.61	84.58 ± 13.22	0.412
Total cholesterol (mmol/l)	4.20 ± 1.11	4.20 ± 1.19	4.21 ± 0.96	0.957
Triglyceride (mmol/l)	1.40 (1.02–1.89)	1.35 (1.00–1.85)	1.46 (1.07–1.95)	0.333
LDL-C (mmol/l)	2.38 ± 0.94	2.38 ± 1.00	2.39 ± 0.82	0.918
Fasting glucose (mmol/l)	5.44 (5.16–6.94)	5.38 (5.11–6.74)	5.83 (5.24–7.49)	0.049*
Blood urea nitrogen (mmol/l)	11.78 (8.21–17.14)	10.89 (8.21–18.12)	13.57 (9.82–16.60)	0.213
Creatinine (µmol/l)	176.80 (128.18–247.52)	172.38 (123.76–247.52)	185.64 (137.02–243.10)	0.296
eGFR (mL/min)	30.98 ± 15.18	32.43 ± 16.13	28.36 ± 13.00	0.066
Total calcium (mmol/l)	2.20 (2.15–2.31)	2.20 (2.13–2.30)	2.22 (2.17–2.32)	0.200
Phosphorus (mmol/l)	1.23 ± 0.26	1.24 ± 0.27	1.20 ± 0.25	0.289
Leptin (ng/ml)	8.79 (5.02–15.90)	7.76 (3.90–13.15)	11.60 (6.26–27.52)	< 0.001*
Female, n (%)	92 (44.9)	62 (47.0)	30 (41.1)	0.418
Diabetes mellitus, n (%)	95 (46.3)	52 (39.4)	43 (58.9)	0.007*
Hypertension, n (%)	170 (82.9)	111 (84.1)	59 (80.8)	0.551
Glomerulonephritis, n (%)	54 (26.3)	39 (29.5)	15 (20.5)	0.161
CCB, n (%)	89(43.4)	52(39.4)	37(50.4)	0.118
ACEi, n (%)	8(3.9)	6(4.5)	2(2.7)	0.523
ARB, n (%)	105(51.2)	67(50.8)	38(52.1)	0.118
Beta blocker, n (%)	61(29.8)	38(28.8)	23(31.5)	0.683
Statin, n (%)	90(43.9)	56(42.4)	34(46.6)	0.566
Fibrate, n (%)	19(9.3)	13(9.8)	6(8.2)	0.700
CKD stage 3, n (%)	99 (48.3)	69 (52.3)	30 (30.3)	0.153
CKD stage 4, n (%)	67 (32.7)	37 (28.0)	30 (41.1)	
CKD stage 5, n (%)	39 (19.0)	26 (19.7)	13 (17.8)	

Values for continuous variables are given as mean ± standard deviation and tested by Student’s t-test; variables not normally distributed are given as median and interquartile range and tested by Mann–Whitney U test; values are presented as number (%) and analysis was done using the chi-square test.

cfPWV, carotid–femoral pulse wave velocity; SBP, systolic blood pressure; DBP, diastolic blood pressure; LDL-C, low-density lipoprotein cholesterol; eGFR, estimated glomerular filtration rate; ARB, angiotensin-receptor blocker; ACE, angiotensin-converting enzyme; CCB, calcium-channel blocker; CKD, chronic kidney disease.

*P < 0.05 was considered statistically significant.

We further analysed multivariable logistic regression for aortic stiffness with adjustment of these 7 variables: DM, age, leptin level, SBP, waist circumference, body fat mass, and fasting glucose. We found that there are three independent predictors of aortic stiffness: age (odds ratio (OR): 1.064, 95% confidence interval (CI): 1.033–1.096, *P* < 0.001), serum leptin level (OR: 1.061, 95% CI: 1.027–1.095, *P* < 0.001), and SBP (OR: 1.021, 95% CI: 1.006–1.037, *P* = 0.006) (Table 2).

**Table 2.** Multivariate logistic regression analysis of the factors correlated to aortic arterial stiffness among 205 chronic kidney disease patients

Variables	Odds ratio	95% confidence interval	*P* value
Leptin, ng/ml	1.061	1.027–1.095	< 0.001*
Age, year	1.064	1.033–1.096	< 0.001*
Systolic blood pressure, mmHg	1.021	1.006–1.037	0.006*
Diabetes mellitus, present	2.111	0.968–4.602	0.060
Body fat mass, %	1.035	0.992–1.079	0.110
Waist circumference, cm	1.013	0.979–1.048	0.449
Fasting glucose, mmol/l	1.012	0.863–1.186	0.886

Analysis data was done using the multivariate logistic regression analysis (adopted factors: diabetes mellitus, age, waist circumference, body fat mass, systolic blood pressure, fasting glucose, and leptin).

*P < 0.05 was considered statistically significant.

Adjusted R^2^ = 0.350. Overall model *P* value < 0.001.

The results of the simple and multivariable linear regression analyses of the clinical variables associated with logarithmically transformed cfPWV (log-cfPWV) values are presented in Table 3. SBP (*r* = 0.392, *P* < 0.001), age (*r* = 0.353, *P* < 0.001), waist circumference (*r* = 0.249, *P* < 0.001), log-leptin level (*r* = 0.237, *P* = 0.001), DM (*r* = 0.203, *P* = 0.003), and diastolic blood pressure (DBP, *r* = 0.156, *P* = 0.026) were positively correlated with log-cfPWV, whereas estimated glomerular filtration rate (eGFR, *r* = −0.184, *P* = 0.008) was negatively correlated. Multivariable forward stepwise linear regression analysis of the factors significantly associated with log-cfPWV revealed four independent predictors of log-cfPWV: SBP (β = 0.352, adjusted *R*^2^ change = 0.150, *P* < 0.001), age (β = 0.308, adjusted *R*^2^ change = 0.0981, *P* < 0.001), log-leptin level (β = 0.192, adjusted *R*^2^ change = 0.042, *P* = 0.001), and DM (β = 0.157, adjusted *R*^2^ change = 0.021, *p* = 0.008).

**Table 3.** Correlation between carotid-femoral pulse wave velocity levels and clinical variables among the 205 chronic kidney disease patients

	Log-cfPWV (m/s)					
	Univariate			Multivariate		
Variables	*r*	*P* value		Beta	Adjusted R^2^ change	*P* value
Female	−0.123	0.080		–	–	–
Diabetes mellitus	0.203	0.003*		0.157	0.021	0.008*
Hypertension	0.058	0.411		–	–	–
Glomerulonephritis	−0.107	0.127		–	–	–
Age (years)	0.353	< 0.001*		0.308	0.098	< 0.001*
Height (cm)	0.035	0.618		–	–	–
Body weight (kg)	0.106	0.130		–	–	–
Body mass index (kg/m^2^)	0.105	0.135		–	–	–
Waist circumference (cm)	0.249	< 0.001*		–	–	–
Body fat mass (%)	0.081	0.249		–	–	–
SBP (mmHg)	0.392	< 0.001*		0.352	0.150	< 0.001*
DBP (mmHg)	0.156	0.026*		–	–	–
TCH (mmol/l)	−0.041	0.560		–	–	–
Log-Triglyceride (mmol/l)	0.084	0.233		–	–	–
LDL-C (mmol/l)	−0.085	0.223		–	–	–
Log-Glucose (mmol/l)	0.112	0.111		–	–	–
Log-BUN (mmol/l)	0.086	0.222		–	–	–
Log-Creatinine (µmol/l)	0.106	0.129		–	–	–
eGFR (ml/min)	−0.184	0.008*		–	–	–
Log-Calcium (mmol/l)	0.069	0.322		–	–	–
Phosphorus (mmol/l)	−0.052	0.459		–	–	–
Log-Leptin (ng/ml)	0.237	0.001*		0.192	0.042	0.001*

Data of carotid–femoral pulse wave velocity, triglyceride, glucose, BUN, creatinine, calcium, and leptin levels showed skewed distribution and therefore were log-transformed before analysis.

Analysis of data was done using the univariate linear regression analyses or multivariate stepwise linear regression analysis (adapted factors were diabetes mellitus, age, waist circumference, SBP, DBP, eGFR, and log-leptin).

cfPWV, carotid–femoral pulse wave velocity; SBP, systolic blood pressure; DBP, diastolic blood pressure; TCH, total cholesterol; LDL-C, low-density lipoprotein cholesterol; BUN, blood urea nitrogen; eGFR, estimated glomerular filtration rate.

*P < 0.05 was considered statistically significant.

## Discussion

Our results revealed among patients with stage 3–5 CKD, higher serum leptin levels, older age, and higher SBP were independent risk factors of aortic stiffness. In addition, higher SBP, older age, higher log-leptin level values, and the presence of DM were positively associated with log-cfPWV values.

Aortic stiffness is a progressive, ageing-related process. The underlying mechanisms include vascular inflammation, activation of the renin–angiotensin–aldosterone system, insulin resistance, and neurohormonal dysfunction[3]. Aortic stiffness is a shared consequence of numerous diseases, including atherosclerosis, hypertension, DM, metabolic syndrome, CKD, and end-stage renal disease[5]. Ectopic fat accumulation in the visceral area induces the secretion of adipokines; this can result in inflammation associated with aortic stiffness [21,22]. Aortic stiffness is characterized by the earlier return of reflected pressure waves from the arterioles towards the heart, which can result in elevated SBP and pulse pressure[23]. A study of a primary care population showed that aortic stiffness was negatively associated with eGFR, with baseline cfPWV and age both being negatively associated with the annual rate of decline in eGFR during long-term follow-up[24]. The pathophysiological factors that contribute to aortic stiffness in patients with CKD include ageing, Framingham risk factors such as DM, impaired glucose tolerance, hypertension, obesity, dyslipidemia, and vascular calcification[6]. Consistent with those findings, the present study showed that DM, age, waist circumference, SBP, and DBP were positively correlated with the log-cfPWV values of patients with CKD, whereas eGFR was negatively correlated. Furthermore, we found that the group of participants with aortic stiffness had a high prevalence of DM, were older, and had higher SBP, after adjusting for the covariates.

Leptin is mainly producing by white adipose tissue [10,25]. It has potentiated many important central and peripheral actions, throughout leptin receptors to regulate energy homoeostasis, fertility, and bone metabolism[11]. In addition, studies have shown that leptin can exert atherogenic, thrombotic, and angiogenic actions related to cardiovascular homoeostasis [10,26,27]. Several reports have proposed possible pathophysiologies underlying the atherogenic and thrombotic effects of leptin. First, leptin may upregulate inducible nitric oxide synthase and thus increase large amount of the production of nitric oxide, which may impair endothelial function and induce atherogenesis by inducing oxidative stress.^11^ Second, many evidence indicate that leptin contribute to thrombosis because of platelet hyperactivity and imbalance between coagulation and fibrinolysis[11]. Third, angiotensin II locally produced by adipocytes may directly increase the secretion of leptin from adipocytes. Both angiotensin II and the leptin from the adipocytes can potentiate sympathetic activity and act synergistically to promote obesity-related hypertension [11,28]. Fourth, leptin has been shown to upregulate inflammatory immune responses by increasing cytokines and growth factors, further inducing atherosclerosis and endothelial dysfunction[29]. Fifth, leptin may enhance the calcification of vascular smooth muscle cells by stimulating osteoblastic differentiation and the production of hydroxyapatite[30]. Finally, leptin can reduce the antioxidative and lipogenic effects of insulin, promoting insulin resistance[31]. All these characteristics of leptin may contribute to aortic stiffness.

Several studies have shown positive associations between hyperleptinemia and cfPWV, including in patients with coronary artery disease and hypertension as well as in patients who have undergone kidney transplantation or who are receiving haemodialysis [17–20]. Moreover, hyperleptinemia has been shown to positively correlate with the degree of coronary artery narrowing, the complexity of atherosclerotic lesions, and the proportion of abnormal coronary artery segments in patients with angiographically diagnosed coronary atherosclerosis[32]. The results of this study showed that after adjusting for confounding factors, hyperleptinemia had positively associated of aortic stiffness and was positively correlated with cfPWV in CKD patients.

This was the first study to focus on the relationship between serum leptin levels and aortic stiffness in patients with stage 3–5 CKD. However, this study has several limitations. First, this study conducted at a single hospital with a limited number of patients with CKD. Second, we did not measure albuminuria in this study. It has been demonstrated that albuminuria is often followed by the development and progression of aortic stiffness and atherosclerosis[33]. Finally, job strain was significantly associated with arterial stiffness in men among enterprise employees from Thailand[34]. In this study, we did not record occupations and labour levels. Further studies are warranted to deduce the cause–effect relationship between serum leptin levels and aortic stiffness in patients with stage 3–5 CKD.

In conclusion, this study investigated the role of leptin in aortic stiffness in patients with stage 3–5 CKD and demonstrated that serum leptin level is positively correlated with cfPWV, indicating a positive association between serum leptin level and aortic stiffness in this patient group.
